# Six-Month Outcomes of Mechanical Thrombectomy for Treating Deep Vein Thrombosis: Analysis from the 500-Patient CLOUT Registry

**DOI:** 10.1007/s00270-023-03509-8

**Published:** 2023-08-14

**Authors:** Abdullah Shaikh, Adam Zybulewski, Joseph Paulisin, Mohannad Bisharat, Nicolas J. Mouawad, Adam Raskin, Eugene Ichinose, Steven Abramowitz, Jonathan Lindquist, Ezana Azene, Neil Shah, James Nguyen, Josh Cockrell, Bhavraj Khalsa, Vipul Khetarpaul, Douglas A. Murrey, Kalyan Veerina, Edvard Skripochnik, Thomas S. Maldonado, Matthew C. Bunte, Suman Annambhotla, Jonathan Schor, Herman Kado, Hamid Mojibian, David Dexter, the CLOUT Investigators

**Affiliations:** 1 Allegheny Health Network Research Institute, 4 Allegheny Square East, Pittsburgh, PA 15212 USA; 2 Mount Sinai Medical Center of Florida, Miami, FL USA; 3 Ascension Genesys Hospital, Grand Blanc, MI USA; 4 HCA Florida Memorial Hospital, Jacksonville, FL USA; 5 McLaren Health System, Bay City, MI USA; 6 Mercy Health - The Heart Institute, Cincinnati, OH USA; 7 Oklahoma Heart Institute, Tulsa, OK USA; 8 MedStar Health Research Institution, Washington, DC USA; 9 University of Colorado, Denver, CO USA; 10 Gundersen Health, La Crosse, WI USA; 11 Aurora St. Luke’s Medical Center, Milwaukee, WI USA; 12 Manatee Memorial Hospital, Bradenton, FL USA; 13 Alabama Clinical Therapeutics, Birmingham, AL USA; 14 Heart and Vascular Center, Providence St. Joseph Hospital, Orange, CA USA; 15 Washington University, St. Louis, MO USA; 16 Inland Imaging at Providence Sacred Heart Medical Center, Spokane, WA USA; 17 Opelousas General, Opelousas, LA USA; 18 Columbia University Irving Medical Center, New York, NY USA; 19 NYU Langone Medical Center, New York, NY USA; 20 Saint Luke’s Mid-America Heart Institute, Kansas City, MO USA; 21 Longstreet Clinic, Gainesville, GA USA; 22 Northwell Health, Staten Island University Hospital, Staten Island, NY USA; 23 William Beaumont Hospital, Royal Oak, MI USA; 24 Yale School of Medicine, New Haven, CT USA; 25 Sentara Vascular Specialists, Norfolk, VA USA

**Keywords:** Deep vein thrombosis, Mechanical thrombectomy, Postthrombotic syndrome

## Abstract

**Purpose:**

Mechanical thrombectomy for the treatment of deep vein thrombosis (DVT) is being increasingly utilized to reduce symptoms and prevent postthrombotic syndrome (PTS), but more data on clinical outcomes are needed. Mechanical thrombectomy was studied in the ClotTriever Outcomes (CLOUT) registry with 6-month full analysis outcomes reported herein.

**Materials and Methods:**

The CLOUT registry is a prospective, all-comer study that enrolled 500 lower extremity DVT patients across 43 US sites treated with mechanical thrombectomy using the ClotTriever System. Core-lab assessed Marder scores and physician-assessed venous patency by duplex ultrasound, PTS assessment using Villalta score, venous symptom severity, pain, and quality of life scores through 6 months were analyzed. Adverse events were identified and independently adjudicated.

**Results:**

All-cause mortality at 30 days was 0.9%, and 8.6% of subjects experienced a serious adverse event (SAE) within the first 30 days, 1 of which (0.2%) was device related. SAE rethrombosis/residual thrombus incidence was 4.8% at 30 days and 8.0% at 6 months. Between baseline and 6 months, venous flow increased from 27.2% to 92.5% of limbs (*P* < 0.0001), and venous compressibility improved from 28.0% to 91.8% (*P* < 0.0001), while median Villalta scores improved from 9.0 at baseline to 1.0 at 6 months (*P* < 0.0001). Significant improvements in venous symptom severity, pain, and quality of life were also demonstrated. Outcomes from iliofemoral and isolated femoral-popliteal segments showed similar improvements.

**Conclusion:**

Outcomes from the CLOUT study, a large prospective registry for DVT, indicate that mechanical thrombectomy is safe and demonstrates significant improvement in symptoms and health status through 6 months.

*Level of Evidence* 3: Non-randomized controlled cohort/follow-up study.

**Electronic supplementary material:**

The online version of this article (10.1007/s00270-023-03509-8) contains supplementary material, which is available to authorized users.

## Introduction

Lower extremity deep vein thrombosis (DVT) is a potentially debilitating disease that can lead to postthrombotic syndrome (PTS), which can involve chronic pain, venous leg ulcers, and long-term disability [1]. The standard of care for acute DVT is anticoagulation (AC)[2]. However, AC alone may be ineffective, as up to 50% of DVT patients develop PTS [1]. Thrombolytic therapies, including catheter-directed thrombolysis (CDT), may be effective in treating DVT and reducing the risk of PTS. Contraindications to thrombolysis, periprocedural bleeding risks [3], and ineffective use of thrombolysis in older thrombus may limit the utility of this therapy [4]. Furthermore, PTS develops at a higher rate in patients with residual venous obstruction [5]. Therefore, treatment options are needed that maximize the balance of complete or near complete thrombus resolution with an acceptable bleeding risk to mitigate long-term complications of DVT.

The ClotTriever System (Inari Medical, Irvine, CA) is a mechanical thrombectomy device indicated for the treatment of DVT. The ClotTriever Outcomes (CLOUT) registry prospectively enrolled 500 patients of any DVT symptom duration to study the safety and effectiveness of this mechanical thrombectomy treatment. The analysis of in-hospital outcomes of all 500 CLOUT patients [6] and an interim analysis of 6-month outcomes in the first 250 patients [7] evaluated the safety and effectiveness of the ClotTriever System. Herein, the 30-day safety profile and clinical outcomes including duplex ultrasound and disease severity assessments through 6 months for the completely enrolled cohort of the CLOUT registry are reported.

## Methods

### CLOUT Registry

CLOUT (NCT03575364) is a prospective, multi-center post-approval study designed to evaluate real-world outcomes following treatment of lower extremity DVT using mechanical thrombectomy sponsored by Inari Medical (Irvine, CA). All patients provided informed written consent pre-procedure and investigators obtained institutional review board approval at each site prior to enrolling patients.

### Patient Population

Patients enrolled in the study were at least 18 years old and had a proximal lower extremity DVT involving at least the femoral, common femoral, iliac veins, or inferior vena cava (IVC), alone or in combination. Exclusion criteria included prior venous stent in a target vessel segment, IVC filter in place at time of thrombectomy, contraindication to anticoagulation, and life expectancy < 1 year. Patients were included regardless of unilateral or bilateral disease, recent failed treatment of the index DVT, or symptom duration.

### Device and Procedure

The ClotTriever System is an over-the-wire mechanical thrombectomy device consisting of a thrombectomy catheter with a nitinol coring element and collapsible bag to core and collect the thrombus for distal protection (Supplemental Fig. S1A), and a 13F or 16F sheath, both of which were available for use in CLOUT, with an expandable funnel to receive and remove the thrombectomy catheter and collected thrombus (Supplemental Fig. S1B). The device can be cleaned and reinserted for additional passes.

Patient and procedural characteristics were collected at the time of diagnosis and during the thrombectomy procedure. Medical history, physical exam including CEAP score [8], anticoagulation regimen, duplex ultrasound (DUS), and health status assessments were performed ≤ 7 days prior to the index procedure or after the symptom onset in those subjects with symptom duration < 7 days.

Intravascular ultrasound imaging was strongly encouraged before and after thrombectomy. Balloon angioplasty and stenting were permitted at the treating physician's discretion. Following treatment with the study device, adjunctive thrombectomy was allowed, including thrombolytic therapy or further mechanical thrombectomy via a different device.

### Safety and Clinical Outcomes

Serious adverse events (SAEs) were independently adjudicated by a medical monitor (InRoad Medical, Honolulu, HI) and defined according to ISO 14155 [9]. The SAEs were attributed to the study device or procedure when appropriate.

Independent core lab-assessed (NAMSA, New York, NY) Marder scores were calculated using intra-procedural venograms obtained before and after treatment, including after venoplasty and/or stenting, if performed [6, 7, 10]. Duplex ultrasound was performed on target lesions at baseline, 30 days, and 6 months post-procedure and was evaluated per each site’s standard procedures. Compressibility by DUS was graded as normal, partial, incompressible, or not evaluable, while flow was designated as present, absent, or not evaluable. Patency was defined as the presence of both flow and normal/partial compressibility.

Villalta scores were obtained by investigators at baseline prior to the procedure, and at 30 days and 6 months post-procedure. The Villalta score assesses 5 patient-symptoms and 6 physician-assessed clinical signs, each on a scale of 0–3, for a final combined score range of 0–33. Patients with PTS were defined as those with a Villalta score at 6 months > 4, and Villalta score/PTS severity was categorized based on Villalta scores as mild (5–9), moderate (10–14), or severe (≥ 15 or presence of venous ulcers) [11].

Additional measures of disease progression and quality of life were collected by investigators at baseline, 30 days, and 6 months. The revised venous clinical severity score (rVCSS) assesses several characteristics of venous clinical presentation and patient parameters, including pain, edema, pigmentation, and ulceration, for an overall score of 0–30 [12, 13]. The numeric pain rating scale (NPRS) is a patient’s verbal self-assessment of pain on a scale of 0 to 10. The EuroQoL group 5-D (EQ-5D) quality of life (QoL) assessment evaluates five aspects of patient status: mobility, self-care, activity, pain, and anxiety, resulting in a scale from 0.0 (equivalent to death) to 1.0 (best possible health) [14, 15].

### Statistical Analysis

Baseline and outcome metrics were summarized using descriptive statistics. Categorical variables were reported as counts with percentages, and continuous variables as medians with interquartile ranges (IQRs). Wilcoxon signed-rank test and McNemar’s or McNemar-Bowker’s tests were applied to test the changes from baseline for continuous and categorical outcomes, respectively, using available paired values. *P* values < 0.05 were considered significant for hypothesis testing. Freedom from rethrombosis/residual thrombus through 6 months was assessed using Kaplan–Meier analysis. Statistical analyses were performed with SAS 9.4 (SAS Institute Inc., Cary, NC) and R 4.0.4 (RStudio Inc., Boston, MA) [16].

## Results

The CLOUT Registry enrolled 500 patients from 43 US sites treated with the study device from September 2018 to February 2022. One patient was determined post-enrollment to have been on hospice with a terminal malignancy and < 1 year expected lifespan at the time of treatment, which was an exclusion criterion. Thus, the patient was removed from the analysis, making the analysis population 499 patients with 521 treated limbs.

### Baseline and Procedural Outcomes

Baseline characteristics and procedural outcomes of the first 500 patients have been previously reported [6] and are summarized here (Table 1). Briefly, the median age of these 499 patients was 61.9 years and 49.5% (247/499) were male. Contraindication to thrombolytics was seen in 29.9% (149/498) of patients. Only 2 limbs were treated with catheter-directed thrombolytics and 2 with other mechanical thrombectomy devices. Venoplasty was performed in 72.7% of limbs, stents were placed in 44.3% of limbs, and IVC filters were placed in 1.4%. Additional key baseline and procedural characteristics are summarized in Table 1.

**Table 1:** Key baseline and procedural characteristics of 499 patients enrolled in the CLOUT registry*

Characteristics	Median [IQR], or *n* (%)
Baseline characteristics	
Age (years)	61.9 [48.0–70.8], *n* = 499
Male sex	247/499 (49.5%)
*Race* (*n* = 488)^†^	
White	376 (77.0%)
Black	101 (20.7%)
American Indian or Alaskan Native	5 (1.0%)
Asian	3 (0.6%)
Other	5 (1.0%)
BMI (kg/m^2^)	30.2 [25.8 – 35.1], *n* = 492
*Symptom duration*^ǂ^	
< 7 days	251/500 (50.2%)
7 – 14 days	121/500 (24.2%)
2 – 4 weeks	70/500 (14.0%)
4 – 6 weeks	23/500 (4.6%)
> 6 weeks	35/500 (7.0%)
Prior history of DVT	124/498 (24.9%)
Contraindication to thrombolytic drug therapy	149/498 (29.9%)
CEAP Score ≥ 3^ǂ^	443/478 (92.7%)

Procedural outcomes	
Single session	496/499 (99.4%)
Thrombectomy procedure time (minutes)	26.0 [18.0 – 40.0], *n* = 464
Estimated blood loss (mL)	40 [20.0, 55.0], *n* = 448
IVUS use^ǂ^	491/521 (94.2%)
*Use of adjunctive treatments*^*ǂ*^	4/521 (0.8%)
Catheter-directed thrombolysis	2/521 (0.4%)
Percutaneous mechanical thrombectomy (other than ClotTriever)	2/521 (0.4%)
Venoplasty^ǂ^	379/521 (72.7%)
Stent placement^ǂ^	231/521 (44.3%)
IVC filter use	7/499 (1.4%)
*Thrombus chronicity (based on most chronic extracted thrombus appearance*^*§*^*)*	
Acute	153/514 (29.8%)
Subacute	177/514 (34.4%)
Chronic	184/514 (35.8%)

^*^See Dexter, et al., (6) for further details

^†^Some patients may check more than 1 race, so numbers will not add up to 488

^ǂ^ Based on # of limbs

^§^Acute: Soft, dark red, and jelly-like; Chronic: firm, fibrous, and pale; Subacute: between acute and chronic

*IQR* interquartile range; BMI: body mass index, *DVT* deep vein thrombosis, *IVC* inferior vena cava

### Anticoagulant Regimen and Compliance

The type of anticoagulant medication prescribed to each patient was left to physician discretion. The types of prescribed anticoagulants reported at discharge and at the 30-day and 6-month visits are presented in Table 2. The majority of patients (81.8%) were discharged with novel/dual acting oral anticoagulants, 6.3% received vitamin K antagonists, and 11.9% reported receiving heparin. The percentage of patients reporting compliance was 94.5% and 94.9% at 30 days and 6 months, respectively.

**Table 2:** Summary of prescribed anticoagulant regimen at follow-ups

Anticoagulant Prescribed	Discharge % (*n*/*N*)	30-day Visit % (*n*/*N*)	6-month Visit % (*n*/*N*)
Heparin, unfractionated	0.9% (4/444)	0.5% (2/390)	0.6% (2/316)
Heparin, low molecular weight	11.0% (49/444)	6.7% (26/390)	4.1% (13/316)
Vitamin K antagonist (VKA)	6.3% (28/444)	5.9% (23/390	8.2% (26/316)
Novel/dual acting oral anticoagulant (NOAC/DOAC)	81.8% (363/444	86.9% (339/390)	87.0% (275/316)
Other/Unknown/None reported/Incomplete data/ Withdrawn/Dead	55	109	183

### Safety Outcomes

**Fig. 1:**
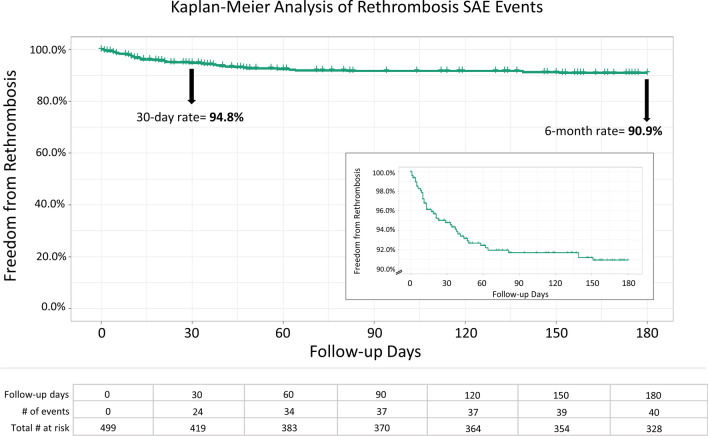
Kaplan–Meier Analysis of Rethrombosis/Residual Thrombus of Treated Vein Segment. Kaplan–Meier analysis of the freedom from rethrombosis events through 180 days is presented, with an abbreviated y-axis (90–100%) shown in the inset. The total number of events and # of patients at risk every 30 days is shown in the chart at the bottom of the figure

Safety outcomes are presented in Table 3. There were 43 SAEs (43/499, 8.6%) over the first 30 days, with 1 device-related SAE (0.2%). Of the 43 SAEs, 24 (24/499, 4.8%) were attributed to rethrombosis/residual thrombus and 4 to pulmonary embolism (PE), with only 1 occurring during the procedure. By 6 months, there were 40/499 (8.0%) rethrombosis/residual thrombus SAEs and no device-related rethrombosis SAEs. Figure 1 demonstrates Kaplan–Meier analysis of rethrombosis events over the first 6 months, yielding freedom from rethrombosis estimate at 30-days of 94.8%, and 90.9% at 6 months. As shown in the inset and the table (Fig. 1), the occurrence of rethrombosis events begins to plateau around 60 days, with only 6 rethrombotic events reported between 60 and 180 days. None of the SAEs reported were related to acute kidney injury or venous valve or vessel damage.

**Table 3:** Key safety events adjudicated by an independent medical monitor

Event	% (*n*/*N*)	Device-related, *n*
All-cause mortality at 30 days	0.9% (4/443)	1
Serious adverse events (% of subjects) at 30 days	8.6% (43/499)	1
Additional key safety events at 30 days^*^		
Rethrombosis/residual thrombus	4.8% (24/499)	0
Pulmonary embolism	0.8% (4/499)	1
Acute kidney injury	0% (0/499)	0
Valve or vessel damage	0% (0/499)	0
Rethrombosis/residual thrombus at 6 months	8.0% (40/499)	0

^*^SAE terms follow Medical Dictionary for Regulatory Activities (MedDRA) terminology

The all-cause mortality through 30 days was 0.9% (4/443), with 1 device-related death. This death was caused by an embolization of IVC thrombus after the catheter became entangled with an embolic protection device, leading to a fatal PE. The 3 additional deaths were due to progression of stage IV non-small cell lung cancer (*n* = 1), a spinal infection following recent lumbar fusion (*n* = 1), and cardiac arrest 11 days post-thrombectomy (*n* = 1).

### Ultrasound Assessments

**Fig. 2:**
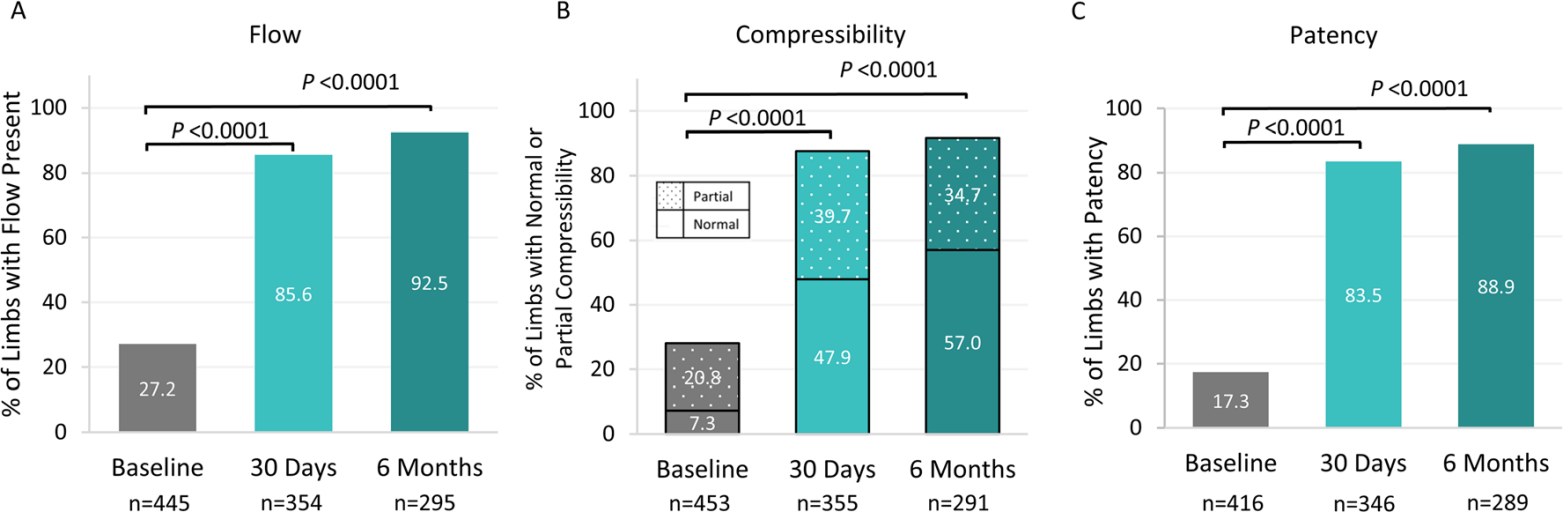
Duplex Ultrasound Outcomes at Baseline, 30 days, and 6 Months A. Percent of limbs with flow present. B. Percent of limbs with normal or partial compressibility. C. Percent of limbs with patency, defined as the presence of flow and normal/partial compressibility

Detectable flow by duplex ultrasound within the affected limb increased significantly from 27.2% at baseline to 85.6% at 30 days and 92.5% at 6 months (Fig. 2A, *P*< 0.0001 for paired values). Similarly, venous compressibility of the treated segments improved from 28.0% at baseline to 87.6% at 30 days and 91.8% at 6 months (Fig. 2B, *P*< 0.0001). Patency increased from 17.3% at baseline to 83.5% at 30 days and 88.9% at 6 months (Fig. 2C, *P* < 0.0001).

### Clinical Outcomes

**Fig. 3:**
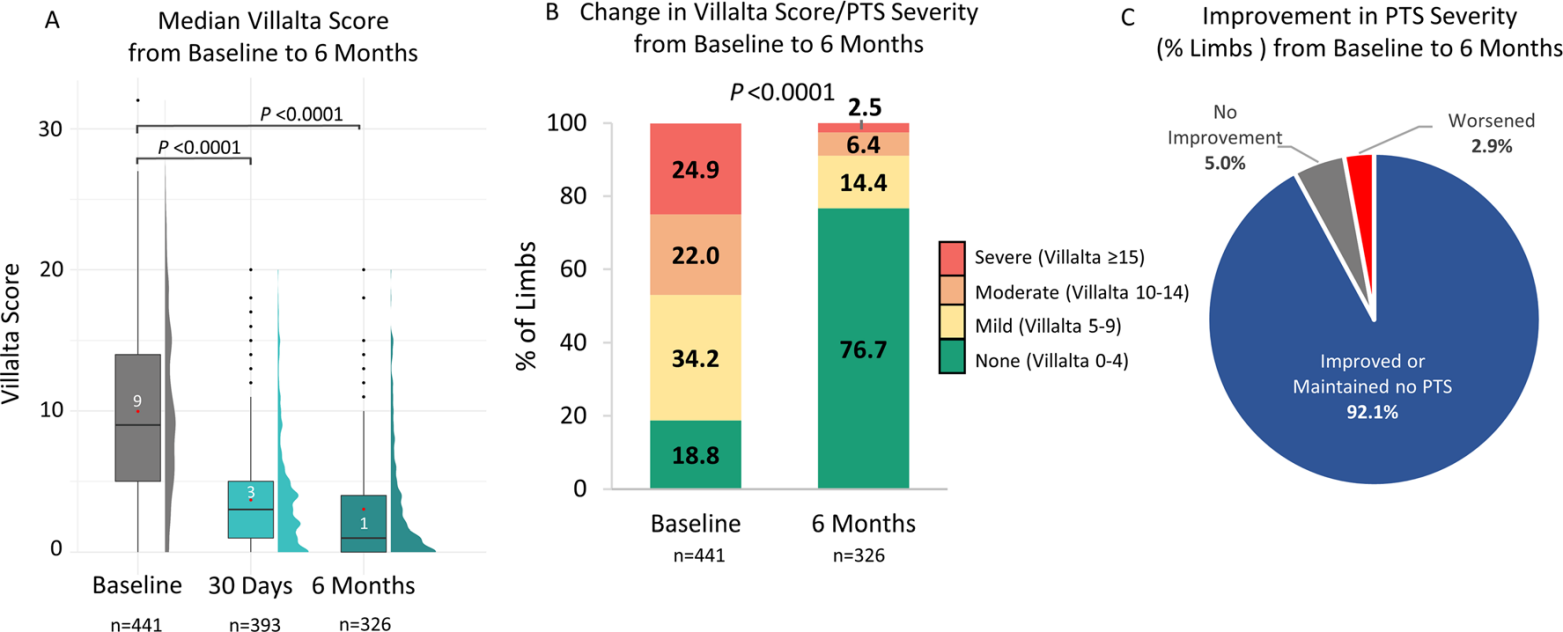
PTS Severity Scores at Baseline to 6 Months. A. Box and half-violin plot of Villalta scores at baseline, 30 days, and 6 months post-procedure. Median is represented by black line; mean is represented by red dot. Black dots are outliers B. Percent of limbs in Villalta score/PTS severity groups at baseline and 6 months post-procedure. Villalta Score/PTS severity categories: No PTS: 0–4; Mild PTS: 5–9; Moderate PTS: 10–14; Severe PTS: ≥ 15. C. Percent of limbs with a change in Villalta score/PTS severity category at 6 months compared to baseline for paired values

Median Villalta score was 9.0 [IQR 5.0, 14.0] at baseline, which improved to 3.0 [1.0, 5.0] at 30 days and 1.0 [0.0, 4.0] at 6 months (*P* < 0.0001), representing improvements of 71.4% and 81.8%, respectively (Fig. 3A, *P *< 0.0001). While 81.2% (358/441) of patients had baseline Villalta score ≥ 5 (defined as PTS when measured at 6 months), this prevalence was reduced significantly to 23.3% by 6 months (76/326; *P* < 0.0001). Similarly, 46.9% (207/441) of patients had baseline Villalta scores ≥ 10 corresponding to moderate-severe PTS, with only 8.9% (29/326) demonstrating the same severity at 6 months (Fig. 3B, P <0.0001). By 6 months, most patients (92.1%) demonstrated improvement in severity category (73.9%) or maintained a Villalta score < 5 (no PTS, 18.2%) at 6 months, whereas 5.0% maintained their PTS severity category, and 2.9% worsened (Fig. 3C).

**Fig. 4:**
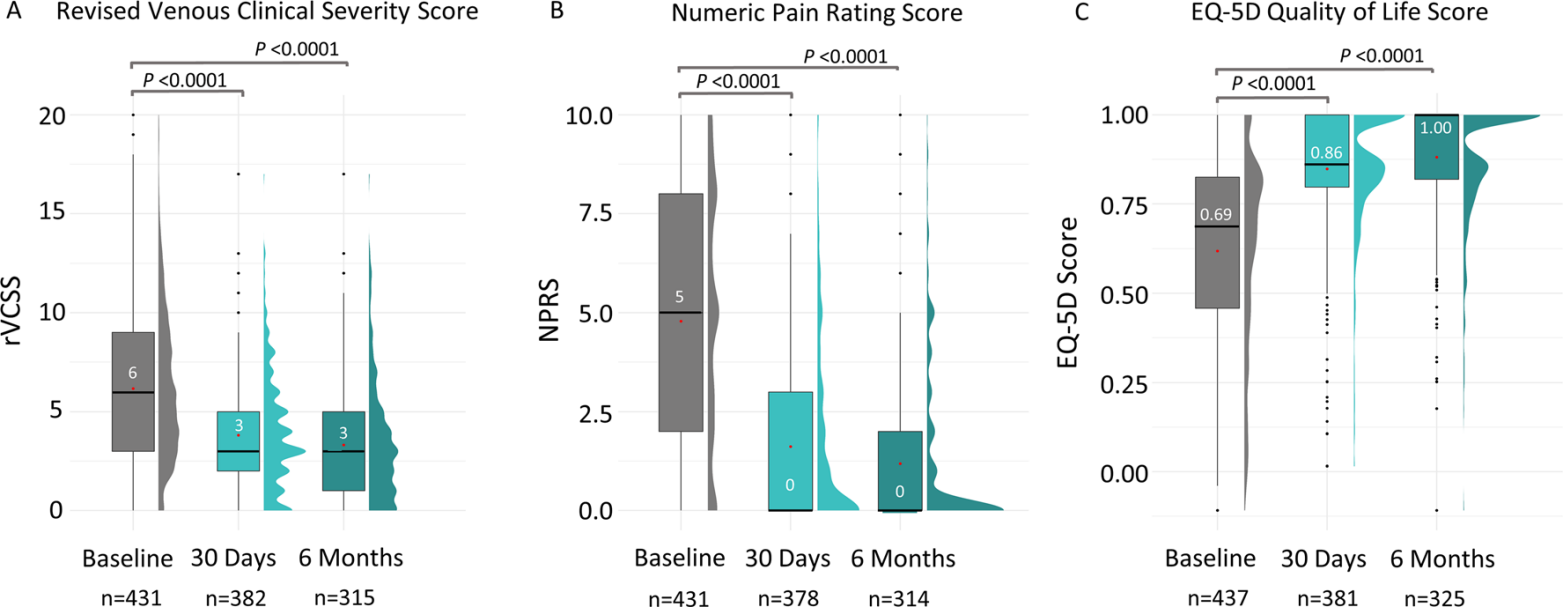
Venous Clinical Severity Score, Pain Score, and Quality of Life Score from Baseline through 6 Months. Clinical outcomes were assessed at baseline, then at 30 days and 6 months post-procedure. Median is represented by a black line; mean is represented by a red dot. A: Revised venous clinical severity score (rVCSS, 0–30 scale); B: Numerical pain rating scale (NPRS, 0–10 scale); C: EuroQoL group 5-D (EQ-5D) quality of life measurement (1 = highest quality)

Significant improvements in several additional clinical parameters were also achieved by 30 days post-procedure and sustained or further improved through 6 months. Improvements in rVCSS were observed, with scores decreasing from a baseline median of 6.0 [3.0, 9.0] to 3.0 at both 30 days and 6 months (Fig. 4A *P* < 0.0001; IQRs [2.0, 5.0] and [1.0, 5.0], respectively). Patients’ pain was also significantly reduced, with median NPRS scores decreasing from 5.0 [2.0, 8.0] at baseline to 0.0 at 30 days and 6 months (Fig. 4B *P* <0.0001; IQRs [0.0, 3.0] and [0.0, 2.0], respectively). Quality of life, measured by the EQ-5D QoL survey, was also significantly improved from a median baseline score of 0.686 [0.458, 0.825] to 0.861 [0.797, 1.000] at 30 days and 1.000 [0.819, 2.000] at 6 months (Fig. 4C *P *<0.0001).

### Iliofemoral and Isolated Femoral-popliteal Analysis

While inclusion criteria did not include patients with only popliteal DVT, many patients had popliteal involvement along with other venous locations of their DVTs. To determine if outcomes differed by initial thrombus location, a sub-analysis of the 381 patients (392 limbs) with any iliac/common femoral (IF) vein versus isolated femoral-popliteal (fem-pop) involvement (108 limbs) of their DVT was undertaken. Among the IF patients, 82.1% (275/335) reported baseline Villalta scores ≥ 5, which improved to 23.5% (57/243) at 6 months, while these proportions were 84.3% and 23.2%, respectively, for fem-pop patients (Table 4). Similarly, rVCSS, NPRS, and EQ-5D scores all demonstrated highly significant improvements from baseline to 6 months for both groups (Table 4; *P* < 0.0001 for each baseline to 6-month comparison).

**Table 4:** Iliofemoral and isolated femoral-popliteal DVT sub-analysis

Characteristic	Median [IQR], or n (%)			
	Iliofemoral (*n* = 381)		Isolated Femoral-popliteal (*n* = 108)	
Age (years)	61.3 [46.9, 70.6]		62.1 [52.5–71.3]	
Male	178/381 (46.7%)		65/108 (60.2%)	
Bilateral DVT	21/381 (5.5%)		6/108 (5.6%)	
Provoked DVT*	164/389 (42.2%)		36/106 (34.0%)	
Contraindicated to thrombolytics	120/380 (31.6%)		27/108 (25.0%)	
Prior history of DVT	95/380 (25.0%)		27/108 (25.0%)	
Previous treatment of current DVT*	91/391 (23.3%)		28/108 (25.9%)	
Marder score, pre-procedure	9.50 [6.75, 13.00]		7.50 [5.00, 8.75]	
Marder score, post-procedure	0.00 [1.00. 1.25]		0.00 [0.00, 1.00]	

	Iliofemoral		Isolated Femoral-popliteal	
	Baseline	6 Months	Baseline	6 Months
Villalta score^†^	9.0 [5.0, 14.0]	1.0 [0.0, 4.0]	10.0 [6.0, 15.0]	2.0 [0.0, 4.0]
PTS^†^	275/335 (82.1%)	57/243 (23.5%)	75/89 (84.3%)	16/69 (23.2%)
rVCSS^†^	6.0 [3.0, 9.0]	3.0 [1.0, 4.0]	6.0 [4.0, 9.0]	3.0 [1.0, 6.0]
NPRS^†§^	5.0 [2.0, 8.0]	0.0 [0.0, 1.0]	5.0 [2.0, 8.0]	0.0 [0.0, 2.0]
EQ-5D^†§^	0.682 [0.458, 0.820]	1.000 [0.803, 1.000]	0.689 [0.456, 0.827]	1.000 [0.833, 1.000]

^*^Based on # of limbs

^†^ *P*-value for statistical comparison between baseline and 6 months was *P* < 0.0001

^§^ Bilateral subjects with clot in iliofemoral segments in only one leg were excluded in these two calculations since the outcomes may be impacted by the other leg with clot in isolated femoral-popliteal. Data from 371 subjects were included in these analyses

*DVT* deep vein thrombosis; *IQR* interquartile range; *PTS* post thrombotic syndrome; *rVCSS* Revised venous clinical severity score; *NPRS* Numerical pain rating scale); *EQ5D* EuroQoL group 5-D quality of life measurement (1 = highest quality)

## Discussion

As a large prospective study of mechanical thrombectomy for DVT, CLOUT offered a uniquely pragmatic enrollment with no exclusions for thrombolytic contraindications or symptom duration. While previously published primary findings from CLOUT demonstrate effective core lab-assessed thrombus removal [6, 7], this current study shows that the effective thrombus removal with selective venoplasty and stenting was accompanied by a robust safety profile with significant and sustained clinical improvements through 6 months. First, the safety profile was promising with a low device-related SAE rate through 30 days. Second, thrombectomy was effective, with excellent Marder score improvements immediately and 88.9% of limbs demonstrating venous patency at 6 months. Third, thrombectomy provided continued improvement in symptoms as most patients (73.9%) showed an improvement in PTS category at 6 months, while another 18.2% remained in the 0–4 Villalta score (no PTS) category. Only 8.9% of patients reported moderate-severe PTS at 6 months. Last, other markers of health status, including the rVCSS, pain scores, and EQ-5D all significantly improved at 6 months.

### Safety Profile

Thirty-day safety outcomes following mechanical thrombectomy were generally favorable, with 1 (0.2%) device-related SAE, 4 (0.8%) PEs, 24 (4.8%) rethrombosis/residual thrombus events, and 0.9% all-cause mortality. The low incidence of PE is notable in light of the infrequent use of IVC filters (8/521, 1.5%, Table 3). No acute kidney injury, or vein or valve damage events, and no major bleeding events were reported. By comparison, while dose reductions in thrombolytics used during CDT are acknowledged [17], bleeding risk remains. From a National Inpatient Sample analysis, the risk of intracranial hemorrhage in CDT-treated patients was 0.7% and significantly higher than the rate of 0.2% in AC alone [18]. In a 2021 meta-analysis, 6.7% of DVT patients receiving CDT experienced bleeding complications compared to 2.2% of patients receiving AC alone, although most bleeding events were seen in older studies [19]. Furthermore, devices utilizing rheolytic mechanisms for thrombectomy can lead to hemolysis and associated acute kidney injury in up to 13% of patients [20–22]. The non-thrombolytic, non-rheolytic, mechanical mechanism of the ClotTriever System may provide a safe and effective treatment alternative with fewer complications.

Rates of rethrombosis/residual thrombus in the full CLOUT study population were 4.8% at 30 days and 8.0% at 6 months. This safety event can occur for a number of reasons unrelated to the study device or procedure, including insufficient stenting, non-compliance with post-treatment AC regimen, or genetic predisposition. The rates in this study are consistent with those found in other DVT studies, including 12% and 8% rethrombosis rates at 24 months in the CDT and AC groups of ATTRACT, respectively [23]. In the CAVA study, recurrence incidence at 12 months was 19.5% for the CDT group, while the standard therapy group experienced 5% recurrence [24]. The CAVENT study reported recurrent DVT incidence at 24 months of 11% in the CDT group and 18% in the standard treatment group [25]. The occurrence of only a few additional rethrombosis events beyond 60 days in the K–M analysis (Fig. 1) provides further evidence of sustained benefit following thrombus extraction.

### Postthrombotic Syndrome

Complete removal of DVT thrombus burden may be critical for assuring positive long-term outcomes, as residual thrombotic deep venous obstruction is associated with adverse outcomes including increased risk of PTS [26–30]. The six-month PTS rate in ATTRACT was 27% in the CDT arm, and 40% in control group, while the 24 months rates were > 40% for both groups [23]. In CAVA, 29% of CDT patients had PTS at 12 months compared to 35% of control group (not statistically different) [24]. On the other hand, CaVenT showed that at least 40% of patients developed PTS at 18 months (41% of CDT group and 56% in the control group) [25, 31]. While it is challenging to compare outcomes across studies, the PTS prevalence at 6 months in the CLOUT registry compares favorably despite the inclusion of patients with any symptom duration, with 23.3% of patients demonstrating PTS, and only 8.9% presenting with moderate-to-severe PTS.

### Iliofemoral and Isolated Femoral-popliteal Patients

Results from the ATTRACT trial demonstrated that CDT provided benefit of reduced PTS only in iliofemoral patients, but not in those patients with isolated femoral-popliteal thrombus [32–34]. Interestingly, the sub-analysis of the patient cohort by thrombus location from the CLOUT registry show that the isolated femoral-popliteal thrombus and iliofemoral subgroups had similar baseline disease severity (Villalta scores, rVCSS, and pain), and they demonstrated similar improvements in these parameters at 6 months post-procedure. More study is needed, particularly with randomized controlled trials, to understand which patient populations will benefit from endovascular treatment over standard AC therapy.

## Study Limitations

The CLOUT registry has several important limitations. The registry was a single-arm device specific study with no comparator group to assess the relative effectiveness of the ClotTriever System. Significant discretion was offered to the investigators regarding adjunctive therapies and anticoagulation, potentially contributing variability. Moreover, flow and compressibility were collected for the entire limb and not per vessel segment, limiting the analysis. Analysis of subgroups, including those with femoral popliteal DVT, were post-hoc analyses without an appropriately powered sample size. Unanticipated deviations from follow-up protocols resulted from restrictions related to the COVID-19 pandemic, which may have introduced inconsistency in reporting late outcome measures.

## Conclusions

The CLOUT registry demonstrated favorable safety and effectiveness for patients through 6 months who underwent thrombectomy using the ClotTriever System. Most patients in this broad population demonstrated improvement in PTS category and other markers of health status through 6 months, with additional long-term follow-up of the study cohort to continue through 2 years. The CLOUT registry will help inform future comparative effectiveness studies, such as the recently initiated DEFIANCE randomized controlled trial, comparing mechanical thrombectomy to standard AC treatment for iliofemoral DVT patients through 6 months.

### Electronic supplementary material

Below is the link to the electronic supplementary material.

Supplemental Figure 1 The ClotTriever System Components and Pre- and Post-procedure Results, A: ClotTriever thrombectomy catheter with a nitinol coring element and integrated collection bag; B: ClotTriever sheath with expandable funnel; C: Representative venogram images from select patients pre- and post-thrombectomy. Arrow colors (green, blue, yellow) depict the same segment in the chronologically sequential sub-panels of each example. Non-occlusive wall-adherent thrombus is visualized by regions of inconsistent opacity in the contrasted vessel and irregular vessel wall delineation. The % Marder score improvement, as assessed by an independent core lab, is provided for each segment.(TIF 3,667 kb)

Supplementary file 2 (DOCX 22 kb)

## Abbreviations

DVT: Deep vein thrombosis
PTS: Postthrombotic syndrome
CLOUT: ClotTriever Outcomes
SAE: Serious adverse events
AC: Anticoagulation
CDT: Catheter-directed thrombolysis
IVC: Inferior vena cava
DUS: Duplex ultrasound
rVCSS: Revised venous clinical severity score
NPRS: Numeric pain rating scale
EQ-5D: EuroQoL group 5-D
QoL: Quality of life
IQR: Interquartile range
PE: Pulmonary embolism
IF: Iliofemoral
