# Supplementary figures and images for: Six-Month Outcomes of Mechanical Thrombectomy for Treating Deep Vein Thrombosis: Analysis from the 500-Patient CLOUT Registry
Source: Cardiovasc Intervent Radiol. 2023 Aug 14;46(11):1571–80. doi: 10.1007/s00270-023-03509-8 (PMC10615929; doi:10.1007/s00270-023-03509-8)

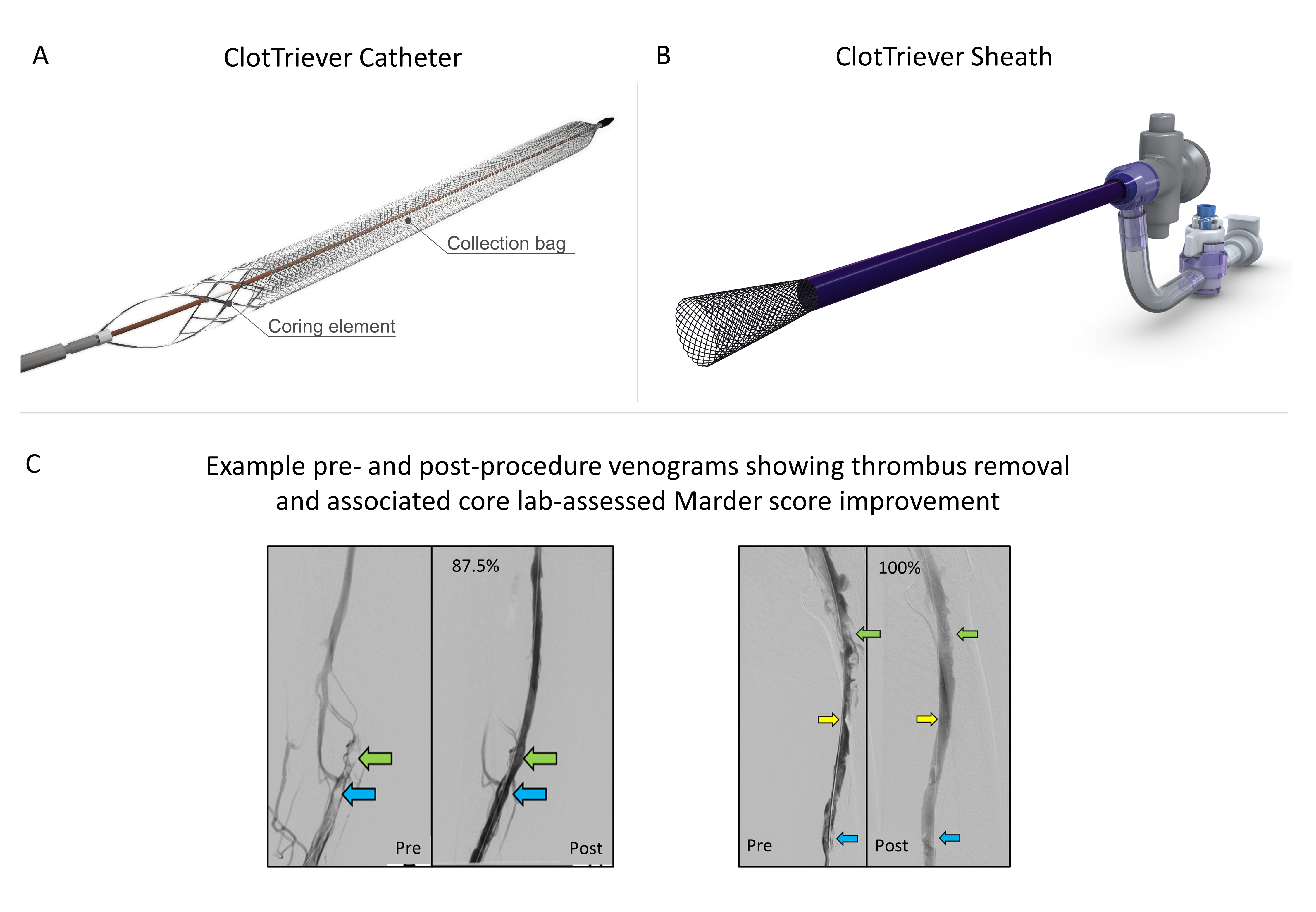

Supplement: Supplementary file 1 — The ClotTriever System Components and Pre- and Post-procedure Results, A: ClotTriever thrombectomy catheter with a nitinol coring element and integrated collection bag; B: ClotTriever sheath with expandable funnel; C: Representative venogram images from select patients pre- and post-thrombectomy. Arrow colors (green, blue, yellow) depict the same segment in the chronologically sequential sub-panels of each example. Non-occlusive wall-adherent thrombus is visualized by regions of inconsistent opacity in the contrasted vessel and irregular vessel wall delineation. The % Marder score improvement, as assessed by an independent core lab, is provided for each segment.(TIF 3,667 kb) [file 270_2023_3509_MOESM1_ESM.tif]
