# Supplementary material for: Six-Month Outcomes of Mechanical Thrombectomy for Treating Deep Vein Thrombosis: Analysis from the 500-Patient CLOUT Registry
Source: Cardiovasc Intervent Radiol. 2023 Aug 14;46(11):1571–80. doi: 10.1007/s00270-023-03509-8 (PMC10615929; doi:10.1007/s00270-023-03509-8)
Supplement: Supplementary file 2 — (DOCX 22 kb) [file 270_2023_3509_MOESM2_ESM.docx]

| **Principal Investigator** | **Site** |
| --- | --- |
| David Dexter (National CoPI) | Sentara Vascular Specialists |
| Robert Beasley (National CoPI),  Adam Zybulewski | Mount Sinai Medical Center of Florida |
| Diego Ayo, Octavio Cosme-Montalvo* | AdventHealth Tampa |
| Christopher King | Affinity Cardiovascular Specialists |
| Joshua Cockrell | Alabama Clinical Therapeutics |
| Jeffery Hnath | Albany Medical College |
| Abdullah Shaikh | Allegheny Health Network Research Institute |
| Joseph Paulisin | Ascension Genesys Hospital |
| Neil Shah | Aurora St. Luke's Medical Center |
| Edvard Skripochnik, Eric Trestman*, Richard Schutzer* | Columbia University Irving Medical Center |
| Ezana Azene | Gundersen Health |
| David O’Connor | Hackensack University Medical Center |
| Eric Trestman | Houston Healthcare Medical Center |
| Ankur Lodha | Lafayette General |
| Fakhir Elmasri | Lakeland Vascular Institute |
| Charles Moomey, Suman Annambhotla* | Longstreet Clinic |
| James Nguyen | Manatee Memorial Hospital |
| Nicolas Mouawad | McLaren Bay Heart and Vascular |
| Steven Abramowitz | MedStar Health Research Institute |
| Mohannad Bisharat | Memorial Hospital Jacksonville |
| Adam Raskin | Mercy Health |
| Wesley Angel, Jon Roberts | Methodist Healthcare Foundation |
| Herman Kado | Millennium Cardiology |
| Jonathan Schor, Saqib Zia* | Northwell Health |
| Thomas Maldonado | NYU Langone Medical Center |
| Eugene Ichinose | Oklahoma Heart Institute |
| Kaylan Veerina | Opelousas General |
| Sagar Gandhi | Prisma Health Upstate |
| Douglas Murrey | Providence Sacred Heart |
| Matthew Bunte | Saint Luke’s Hospital of Kansas City |
| Bhavraj Khalsa | St. Joseph Hospital, Vascular Specialists of OC |
| Matthew Jung | Surgical Care Associates |
| Daniel Long | The Christ Hospital |
| Robert Ford, Ronald Winokur* | Thomas Jefferson University |
| Sonya Noor | SUNY, The University of Buffalo |
| Adam Plotnik | University of California, Los Angeles |
| Jonathan Lindquist | University of Colorado |
| Ambarish Bhat | University of Missouri |
| Stuart Harlin | University of Texas Health Sciences Center at Houston |
| Vipul Khetarpaul, Luis Sanchez* | Washington University |
| Shuo Li | Wesley Medical Center |
| Graham Long | William Beaumont Hospital |
| Hamid Mojibian | Yale University |

* Previous PI
